# Effects of BPZ, BPC, BPF, and BPS Exposure on Adult Zebrafish (*Danio rerio*): Accumulation, Oxidative Stress, and Gene Expression

**DOI:** 10.3390/ijerph192315784

**Published:** 2022-11-27

**Authors:** Ying Han, Yuxuan Liu, Mingxin Wang, Yingang Xue

**Affiliations:** School of Environmental Science and Engineering, Changzhou University, Changzhou 213164, China

**Keywords:** bisphenol analogues, zebrafish, accumulation, antioxidant enzyme, gene expression

## Abstract

As substitutes for bisphenol A (BPA), bisphenol analogs (BPs) have been found to cause endocrine disorders and induce toxic effects. The objective of this study was to evaluate the bioaccumulation and subacute toxicity of bisphenol Z (BPZ), bisphenol C (BPC), bisphenol F (BPF), and bisphenol S (BPS) to zebrafish. Five-month-old zebrafish were exposed to 1/100 LC_50_, 1/50 LC_50_, and 1/10 LC_50_ of BPZ, BPC, BPF, and BPS for 13 days, respectively. Bioaccumulation, oxidative stress, and related mRNA expression in zebrafish tissues were measured on days 1, 7, and 13. After exposure, the four kinds of BPs all resulted in the accumulation of concentration and lipid peroxidation in zebrafish tissues to varying degrees. BPZ and BPC had the highest bioaccumulation level and had the greatest influence on malonic dialdehyde (MDA). In addition, the enzyme activities of superoxide dismutase (SOD), peroxidase (POD), glutathione peroxidase (GSH-PX), and the content of glutathione (GSH) in zebrafish tissues were also affected at different levels. However, the enzyme activities of SOD and POD were inactivated in zebrafish exposed to a high concentration of BPC. Further studies showed that BPs exposure down-regulated the transcription level of *sod* but up-regulated the relative expression levels of *cat* and *gpx*. The mRNA relative expression level of *erα* was not significantly changed, while the mRNA relative expression level of *erβ_1_* was significantly down-regulated except under BPS exposure. These results indicate that BPZ, BPC, and BPF significantly affect the expression level of the estrogen receptor (ER) in zebrafish tissues. Overall, the results suggest that exposure to waterborne BPs can cause severe oxidative stress and tissue damage in adult zebrafish that is not sufficient to kill them after 13 days of waterborne exposure. The toxicity of BPs to organisms, therefore, should be further analyzed and evaluated.

## 1. Introduction

Bisphenol analogs (BPs) are a kind of endocrine disruptor widely existing in water, which are formed by the chemical structure bonding of two hydroxyl phenols. BPs are often used as raw material monomers in plastic production or additives because of their low cost and stable chemical properties. With the restriction of bisphenol A (BPA) in many countries and regions as potential substitutes for BPA, the usage amount of bisphenol Z (BPZ), bisphenol C (BPC), bisphenol F (BPF), and bisphenol S (BPS) have increased significantly [1,2]. Their phenols are all attached to the same carbon atom, and they are insoluble in water. The structures of BPZ, BPF, and BPS are completely symmetrical, while the chemical characteristics of BPZ and BPC are similar. BPZ is used to cure highly heat-resistant plastic materials and in electrical insulation [3]; BPS is widely used in the manufacture of epoxy resin, baby bottles, and thermal paper because of its good thermostability and photostability [4,5]; BPF is mainly used in polycarbonate plastic, varnish, paint, gasket, adhesive, and other products manufacturing [6]; BPC can be used in the preparation of flame retardants [7]. 

As BPs are not easy to be degraded, a large amount of plastic waste without proper treatment will enter the water environment even after sewage treatment and biodegradation, resulting in an increase in BPs content in the water environment [8] and BPs in the environment can enter organisms through contact, diet, and exposure to water. Recent studies have shown that BPs can mimic estrogen activity, compete with endogenous hormones in order to interfere with their normal generation and metabolism of them, and cause the endocrine disorder, which can lead to abnormal reproductive function of the body, such as sexual precocity of the female mammals and the decrease in sperm count in males [9,10,11,12]. They can also weaken the immune system and increase the risk of breast and prostate cancer [13,14]. BPs exposure has also been shown to induce toxic effects such as reproductive toxicity, cytotoxicity, and genotoxicity [15,16,17].

Increasing studies have investigated the toxicity of BPs to organisms. It has been demonstrated that BPZ, BPS, and BPF significantly interfere with the thyroid hormones of embryo-larval zebrafish [18]. In addition, BPF also altered gene transcription of the hypothalamic–pituitary–thyroid axis and systemic thyroid hormone content in embryo-larval zebrafish [19]. BPS can not only inhibit pepsin activity [20] but also affect the feeding behavior of mice and the early development of zebrafish offspring [9,21]. In vitro and in vivo experiments have confirmed that BPS can cause oxidative stress and have anti-androgen properties in rats [22]. Recent studies also revealed that chronic exposure to low concentrations of BPS reduced the transcription of several antioxidant genes, including *gpx1*, *Cu/Zn-sod*, and *cat*, indicating decreased antioxidant capacity and elevated oxidative stress in the adult zebrafish brain [23]. The latest research showed that BPC is more detrimental to theca cell viability and progesterone production compared to BPA [7]. However, to the best of our knowledge, there are a few studies on the toxic effects of mid-long-term exposure to BPs on aquatic life, and they mainly focus on reproductive toxicity and developmental toxicity. When pollutants enter the environment, most substances dilute and spread to a larger area, except a few substances cause acute poisoning and death near the pollution source, and their concentrations gradually decrease, which leads to biological subacute toxic effects. This effect is often latent, slow, and hidden, but it is also very alarming because it will lead to deliberate or nonspecific changes in the biochemistry, physiology, organization, and behavior of organisms, and it is often irreversible. Because these changes are difficult to be found in the early stage, people should pay more attention to the hazards of medium and long-term exposure, especially for aquatic environments and organisms, compared with short-term exposure. A subacute toxicity experiment is based on this situation. Thus, it is very important to study the subacute toxicity of BPs.

Zebrafish are a common model organism. It can be raised on a large scale because of its small size, easy feeding, short life cycle, and high spawning rate. It is easy to be observed because of its clear embryo, rapid development, and external fertilization. At present, zebrafish gene sequencing has been basically completed, and many genes of zebrafish have high homology with humans. The above reasons give zebrafish great advantages in biological and toxicological studies. Therefore, in recent years, zebrafish as an aquatic model organism has been widely used in the monitoring of environmental toxins and used in the cumulative effect and toxicity studies of carcinogens such as harmful heavy metals, pesticides, and phenol [24,25,26].

This study took adult zebrafish as the research object, studied the enrichment capacity of zebrafish tissues exposed to BPZ, BPC, BPF, and BPS, respectively, analyzed the oxidative damage of zebrafish tissues, and detected the expression levels of related genes in tissues after exposure, evaluated the subacute toxic effects of BPZ, BPC, BPF, and BPS respectively. The result provides a reference for the environmental health risk assessment of four emerging BPs and supplements their toxicological data.

## 2. Materials and Methods

### 2.1. Chemicals and Reagents

BPS (CAS: 80-09-1, purity = 99%), BPC (CAS: 14868-03-2, purity >98.0%), and BPZ (CAS: 843-55-0, purity ≥98.0%) were purchased from Shanghai Aldin Reagent Co., Ltd., Shanghai, China. BPF (CAS: 620-92-8, purity = 98%) was purchased from Shanghai Maclin Biochemical Technology Co., Ltd., Shanghai, China. Dimethyl sulfoxide (DMSO) was purchased from Shanghai Lingfeng Chemical Reagent Co., Ltd., Shanghai, China. Isotope internal standard BPS-^13^C_12_(^13^C_12_H_10_O_4_S) (99.0%) and BPA-^13^C_12_ (^13^C_12_H_16_O_2_) (99.0%) were purchased from A ChemTek, Inc., MA, USA. Trizol reagent, dATP, dTTP, dCTP, and dGTP were purchased from Thermo Scientific, Waltham, MA, USA. DNase I was purchased from New England Biolabs Co., Ltd., Ipswich, MA, USA. ABI 7500 system was purchased from Life Technologies Co., Ltd., Carlsbad, CA, USA. SYBR^®^Premix Ex TaqTM II (Perfect Real Time) was purchased from Takara Biomedical Technology (Beijing) Co., Ltd., Beijing, China. TaKaRa AMV Kit was purchased from Takara Biomedical Technology (Beijing) Co., Ltd., Beijing, China. The reagent kits for measuring peroxidase (POD, A084-1-1), malondialdehyde (MDA, A003-1-2), glutathione (GSH, A006-1-1), glutathione peroxidase (GSH-PX, A005-1-2), total protein (TP, A045-2-2) and total superoxide dismutase (T-SOD, A001-1-2) were purchased from Nanjing Jiancheng Bioengineering Institute, Nanjing, China. All chemicals or solvents used in this study were of HPLC grade or analytical grade.

### 2.2. Zebrafish Maintenance and Solutions Preparation

Based on previous experience and in order to avoid the disturbance of oxidative stress during the spawning period, about 1440 AB-type male zebrafish aged 4–5 months were used in this experiment. Their average body length and body weights were 2.42 ± 0.33 cm and 0.16 ± 0.04 g, respectively. The zebrafish were purchased from Jiayu Aquarium (Shanghai, China) and kept in the laboratory. The approval number for breeding and experiment of zebrafish in this study is SCXK-2016-0010. Two weeks in advance, zebrafish used in subsequent experiments were acclimatized in glass tanks filled with aerated 72 h dechlorinated tap water. The pH value, hardness, temperature, and dissolved oxygen of tap water are 7.34–7.62, 95–112 mg/L as CaCO_3_, 24 ± 1 °C, and above 7.2 mg/L, respectively. During domestication, the photoperiod was 14 h light: 10 h dark. The zebrafish were fed twice a day with commercial flake diet at a rate of 5% of body weight. Throughout the domestication period, the dechlorinated tap water was renewed every 2 days, and the cumulative mortality rate of zebrafish was no more than 5%.

DMSO was used as a cosolvent in the experiment. The pre-experiment showed that a low concentration (<0.1%) of DMSO in the zebrafish treatment solution did not affect the experimental results. Four BPs were dissolved with DMSO to be prepared into mother liquor of 1 mg/L (Stored in the fridge at 4 °C, away from light). Immediately before the exposure experiment, they were diluted with DMSO to the corresponding concentration. The final concentration of DMSO in the exposed solution did not exceed 1 mg/L.

### 2.3. Design of the Subacute Toxicity Test

According to Chemicals—Fish, prolonged toxicity (GB/T 21808, 2008), the subacute toxicity of zebrafish exposed to four separate BPs was measured by an experiment of 13 days. On the basis of the acute toxicity of four bisphenol analogs to zebrafish, which has been studied [27], three concentration groups were set up, which were 1/10 LC_50_, 1/50 LC_50_, and 1/100 LC_50_, respectively. The specific concentrations are shown in Table 1. After calculation, the mother liquor was diluted into a corresponding amount of solution and put into a 10 L glass beaker containing 8 L dechlorinated tap water to make exposure solution of the corresponding concentration. Three repetitions were set for each exposure group, and DMSO solvent with the same concentration was set for the control group to avoid interference simultaneously. Each repetition consisted of 30 adult zebrafish and 8 L exposure solution. The semi-static method was used; that is, the water quality conditions were the same as the water used in the domestication process, and the exposure fluid was changed once a day. In addition, in order to prevent the feed from affecting the toxicity sensitivity of zebrafish to BPs, the zebrafish were not fed during the experiment. Meanwhile, dead fish bodies and feces were removed timely to avoid external interference.

### 2.4. Sampling

The euthanasia of zebrafish is based on the current AVMA Guidelines for the Euthanasia of Animals (2013) by America Veterinary Medical Association, which is in line with the relevant principles of animal protection. On days 1, 7, and 13, nine zebrafish were randomly selected from each repetition and frozen in the ice pack immediately. The zebrafish were washed with pre-cooled normal saline and then dried with filter paper and divided evenly into three groups. The first group was quickly frozen in liquid nitrogen after being fixed in 1 mL Trizol reagent and kept at −80 °C for determination of gene expressions. The second group was made into 10% homogenate of muscle tissue, and the supernatant was taken for determination of total protein and enzymatic activities. The last group was stored at −20 °C fridge for total BPs accumulation.

### 2.5. Samples Analysis

#### 2.5.1. Total BPs Accumulation

The concentration of BPs in muscle tissue is an important index to judge the toxic accumulation of zebrafish. Previous experience has shown that the content of BPs in muscle tissue is much higher than that in other organ tissues. Therefore, in order to study the enrichment capacity of muscle tissues of zebrafish exposed to BPZ, BPC, BPF, and BPS, respectively, the concentrations of substances in muscle tissues of zebrafish at the 7th and 13th days of exposure were measured. The treatment procedure of samples included several steps. First, 0.2 g of freeze-dried muscle tissue samples were weighed. Then the samples were added into 6 mL acetonitrile, ground for 2 min, and vortexed for 1 min. At last, they were mixed in a 20 °C-gas bath constant temperature oscillator for 30 min, extracted by ultrasound for 30 min, and centrifuged at 8000 r/min for 10 min (Repeat extraction twice). The extracted solution was combined and placed in a refrigerator at −80 °C for 48 h for fat removal. After removal, the solution was blown by nitrogen to nearly dry and redissolved by 1 mL methanol for U-HPLC analysis.

#### 2.5.2. Biochemical Analysis

In order to measure the content of GSH, MDA and analyze enzyme activities of SOD, POD, and GSH-PX, zebrafish tissue samples were placed in pre-cooled normal saline to make 10% tissue homogenate (1:9, *w*/*v*) and centrifuged with a high-speed refrigerated centrifuge for 10 min in the condition of 8000 r/min at 4 °C. The supernatant was taken and analyzed with commercial kits (Nanjing Jicheng Institute of Biological Engineering, Nanjing, China).

#### 2.5.3. mRNA Expression Analysis

The frozen zebrafish tissue pieces were quickly ground in liquid nitrogen and homogenized in 1 mL Trizol reagent, and total RNA was isolated from those samples according to the manufacturer’s protocol. In order to explore the possible mechanism of BPs exposure in zebrafish, three antioxidant enzyme genes and two estrogen receptors were selected, and their gene expression in zebrafish was evaluated. Among them, the antioxidant enzyme gene corresponds to the enzyme activity measured [16], and the estrogen receptor can reflect the organism’s cellular immunity [11]. Sequences of primers (Table 2) were obtained from Jiangsu Hongzhong Biotech Co., Ltd. After the cRNA was synthesized by reverse transcription, one of them was selected for reference gene amplification verification. Genes with Ct values between 15 and 25 and good repeatability and stability were selected as the internal reference for large-scale qPCR of subsequent samples. qPCR analysis was performed using ABI 7500 system with *β-actin* and *rp17* as internal reference genes. qPCR assays were carried out with a 25 µL reaction volume containing 1 µL of reverse primer, 1 µL of forwarding primer, 12.5 µL of SYBR^®^Premix Ex TaqTM II, 8.5 µL of nuclease-free water, and 2 µL of cDNA template. The qPCR program consisted of initial denaturation at 95 °C for 2 min, followed by 40 cycles of 96 °C for 10 s and 60 °C for 30 s. All sample reactions were performed in triplicates. The relative expression of the target gene was calculated with *rp17* by the 2^−ΔΔCt^ method [28].

### 2.6. Statistical Analysis

Data were collected and sorted out by Microsoft Excel 2018 and statistically described by SPSS 26.0. Experimental results were presented as mean ± standard deviation (SD). The standard deviation was presented by the error bar. The mean of multiple samples was analyzed by one-way ANOVA, and Duncan’s multiple range test was used for pairwise comparison between groups. *p* < 0.05 was defined as statistically significant. The plots were displayed by Origin 2018.

## 3. Results and Discussions

### 3.1. Method Validation

In this study, a series of standard solutions ranging from 0.5 μg/L to 100 μg/L were prepared for the measurement. Draw the standard curve where the ratio of ion peak area between the object to be measured and the internal standard is the ordinate, and the mass concentration of the object to be measured in the sample is the abscissa. BPA-^13^C_12_ was used as the internal standard in BPZ, BPC, and BPF. BPS-^13^C_12_ was used as the internal standard in BPS. The additional amount of internal standard was 50 ng. The limit of detection (LOD) and limit of quantification (LOQ) were determined by adding the target substance to blank samples. According to guideline 203 of OECD, the measured concentration of the tested chemical should be ranged from 80% to 120% of the nominal concentration. As shown in Table 3, the linearity of BPZ, BPC, and BPF was good in the range of 1–100 μg/L, the correlation coefficient(R^2^) was > 0.9989, the LOD was 0.12–0.60 μg/L, and the LOQ was 0.38–1.89 μg/L; the linearity of BPS was good in the range of 0.5–100 μg/L, the correlation coefficient(R^2^) was 0.9990, and the LOD was 0.019 μg/L, the LOQ was 0.06 μg/L. A total of 0.2 g fish samples (500 mL water samples) were accurately weighed, and a 50 ng (20 ng) standard internal mixture was added. The experiment of standard adding recovery was carried out in BPs solution of 1.5, 4.5, and 15 μg/L, respectively. Meanwhile, blank control was made, and each group was performed in parallel six times. The results show that the recovery rate of BPs in fish samples (water samples) ranges from 83.30% to 97.45% (91.45% to 102.91%), and the relative standard deviation is from 4.63% to 16.36% (1.47% to 11.04%). Meanwhile, no BPs were detected in control groups. Therefore, the nominal concentration of different exposure groups in the following tests equated to the actual concentration, and the exposure protocols of BPs for zebrafish were reasonable.

### 3.2. Residual Concentrations of BPs

**Figure 1:**
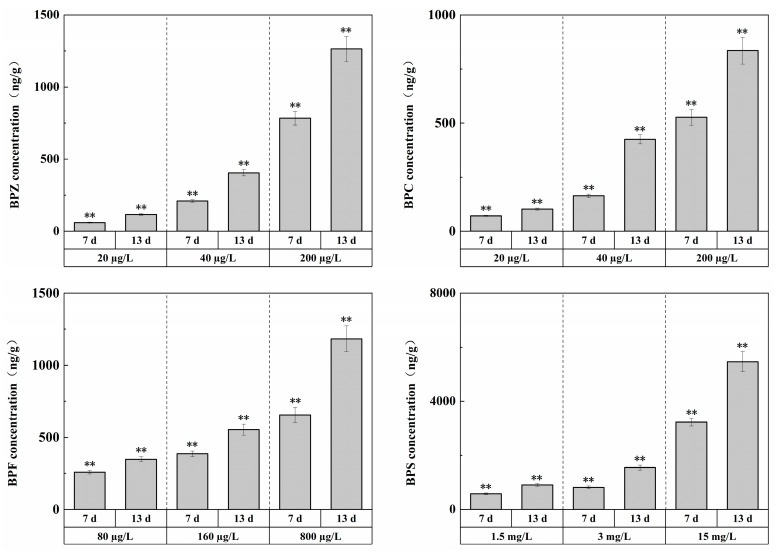
Effects of waterborne BPs exposure on the accumulation in the tissues of zebrafish at different exposure conditions after 7, 13 days. The error bars represent the SD. The asterisks represent significant difference between treatment and control group at the same timepoint (*p* < 0.05, **).

The concentrations of target pollutants in 1/100 LC_50_, 1/50 LC_50_, and 1/10 LC_50_ exposure groups were measured before and after changing the exposure solution every day, and the concentrations of target pollutants remained basically stable during exposure. After exposure for 13 days, the concentration changes of the four substances in zebrafish tissues, respectively, were shown in Figure 1, with the overall level between ng/g and μg/g.

The experiment showed that under exposure to BPZ, BPC, BPF, and BPS, respectively, with the increase in waterborne BPs concentrations, significant BPs accumulation occurred in the tissues. In other words, there was a bio-enrichment effect in zebrafish tissues, and the enrichment level was concentration-dependent; that is, with the increase in exposure duration, the enrichment level showed an increasing trend. This is to be expected, indicating that BPs are difficult to degrade in vivo and that residual concentrations in vivo are highly correlated with exposure levels.

Compared with each other, BPZ had the strongest enrichment ability, while BPS had the weakest. This may be related to hydrophobicity. Studies have shown that hydrophobicity is the main driving force of bisphenol compound accumulation, and the bioaccumulation factor (Log BAF) is significantly positively correlated with Log Kow [29]. In addition, the acute toxicity of the four substances may also be a major factor in the strength of enrichment ability. In this experiment, the number of substances with weak acute toxicity was larger, and the bio-enrichment effect of zebrafish tissues might be stronger [30].

### 3.3. Effects of BPs Exposure on Lipid Peroxidation

MDA is the final decomposition product of lipid peroxidation (LPO) and can affect cell membranes. MDA content is an important parameter reflecting the potential antioxidant capacity of the body, which can reflect the lipid peroxidation rate and intensity of the body and also indirectly reflect the level of tissue peroxidation damage [31]. In addition, antioxidant and detoxifying systems could alleviate LPO by eliminating excess reactive oxygen species (ROS) induced by xenobiotics [32].

**Figure 2:**
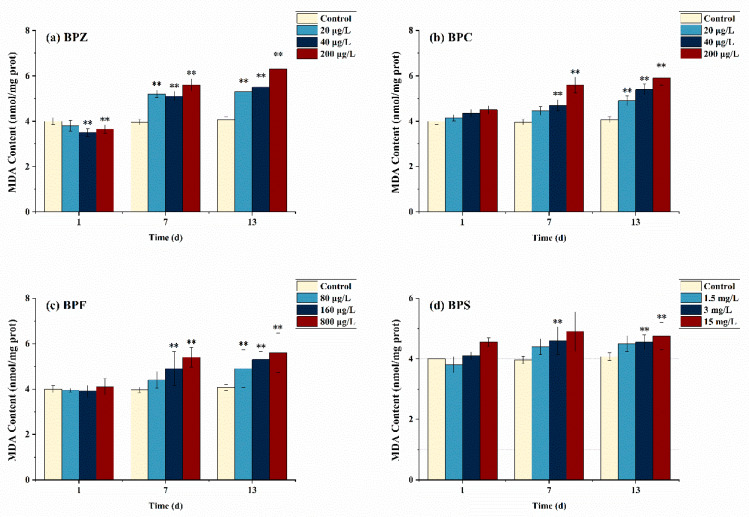
Effects of waterborne BPs exposure on the content of MDA in the tissues of zebrafish under different exposure conditions after 1, 7, and 13 days. The error bars represent the SD. The asterisks represent significant difference between treatment and control group at the same timepoint (*p* < 0.05, **).

Figure 2 describes the effects of BPs on the MDA concentration of adult zebrafish under different exposure conditions. Compared with the control group, MDA levels in all groups except BPZ treatments (40 μg/L and 200 μg/L) did not increase significantly after exposure to BPs for 1 day. However, after BPs exposure for 13 days, MDA levels were significantly increased in all groups, and only the low-concentration BPS treatment group (1.5 mg/L) had no significant difference. Similar results were obtained when the toxicity of BPs was studied in rats, showing that BPS exposure significantly increased rat testicular ROS levels and induced lipid peroxidation [33].

After exposure to BPs for 1 day, MDA content in zebrafish tissues did not significantly increase, which could be interpreted as that the antioxidant system operated effectively and continuously without being too much affected under short-term and low-concentration BPs exposure and eliminated excessive ROS in time. Interestingly, MDA content in tissues increased significantly when the exposure time was extended to 13 days, which is probably because the antioxidant system was damaged and could not remove excessive ROS in time. Consequently, the ROS content exceeds the self-regulated threshold value of zebrafish, thus aggravating the LPO level in vivo [34]. The same phenomenon was observed in BPZ, BPC (40 μg/L and 200 μg/L), and BPF (160 μg/L and 800 μg/L) treatments after exposure to BPs for 7 days. In addition, although not obvious, MDA content stopped rising and even showed a downward trend in the BPS exposure group on day 13, suggesting that the lipid peroxidation level of adult zebrafish exposed to BPS may reach the threshold on day 7. In conclusion, our study showed that after exposure to four kinds of BPs, MDA content increased in adult zebrafish tissues to varying degrees, which led to lipid peroxidation in zebrafish and indirectly reflected the damage of oxygen free radicals on the anti-oxidation system.

### 3.4. Effects of BPs Exposure on Antioxidant Enzyme Activities

It is well known that antioxidant enzymes such as SOD, POD, and GSH-PX play an important role in the antioxidant defense system of cells. Oxidative stress occurs when steady-state ROS levels are temporarily or chronically elevated and can lead to cell damage. SOD-CAT system formed the first line of defense against oxygen toxicity. SOD is the primary material of organisms to remove free radicals. It plays a vital role in the balance between oxidation and anti-oxidation, and the level of its activity in the biological body is an intuitive indicator of aging and death. It can catalyze the superoxide anion into H_2_O_2_ and H_2_O [35]. POD is an enzyme with high activity and widely exists in organisms. It can further transform H_2_O_2_ into H_2_O together with CAT and other enzymes, thus preventing cells from being poisoned by H_2_O_2_. It is also one of the key enzymes in the biological defense system [36]. GSH-PX can catalyze and transport the special toxin-removing substance named GSH, which can not only act as a substrate or cofactor but also directly remove lipid peroxides induced by ROS and hydroxyl groups, thus protecting the integrity of cell membrane structure and function [37].

**Figure 3:**
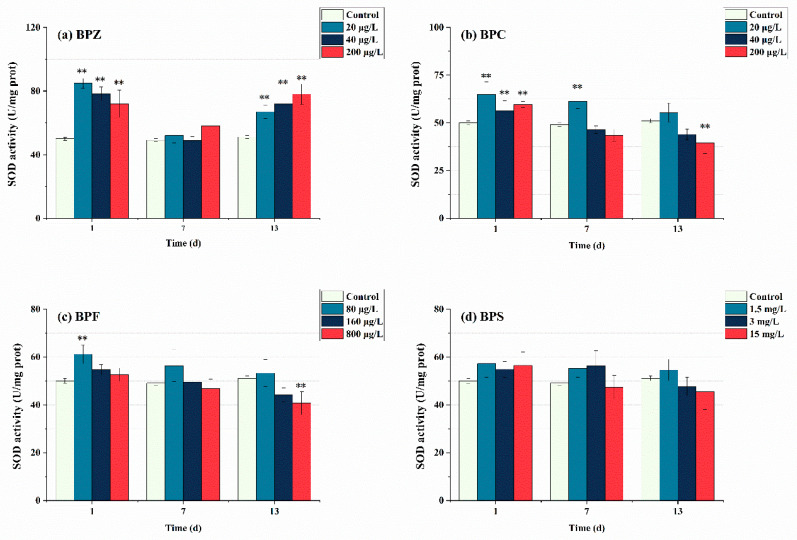
Effects of waterborne BPs exposure on the activity of SOD in the tissues of zebrafish under different exposure conditions after 1, 7, and 13 days. The error bars represent the SD. The asterisks represent significant difference between treatment and control group at the same timepoint (*p* < 0.05, **).

Figure 3 shows the SOD enzyme activity measured on days 1, 7, and 13 after BPs exposure. In this study, after exposure to BPs for 1 day, SOD in each group was significantly induced. The SOD activity of BPZ treatments was even higher than 70 U/mg prot. This indicates that the zebrafish showed oxidative stress reaction when exposed to four BPs, respectively, which promoted the production of SOD in tissues to eliminate the excess ROS, thus forming a protective mechanism against oxidative stress. When the exposure time was extended to 7 days, the SOD activity of the four BPs showed a trend of inhibition, and the SOD activity of BPC (40 μg/L and 200 μg/L) treatments, BPF (800 μg/L) treatment, and BPS (15 mg/L) treatment was lower than that of the control group, which confirmed the previous conclusion that SOD was eliminating ROS to maintain a dynamic equilibrium state of tissues. However, SOD activity decreased faster in the high-concentration exposure group, indicating that the production of ROS is concentration-dependent. When the exposure time lasted up to 13 days, except for BPC (20 μg/L), BPF (80 μg/L), and BPS (1.5 mg/L and 3 mg/L) treatments, SOD activity in other groups were lower than that in the control group, showing inhibition state. It indicated that excessive ROS induced by BPs might inhibit SOD activity, which may exceed the tolerance of SOD. A theory was proposed that the active-site residues of SOD were modified by excessive ROS oxidation, resulting in the loss of enzyme activity and function [38]. Interestingly, SOD activity in the three BPZ exposure treatments was significantly induced again, almost returning to the level after the first day of exposure. This indicates that under exposure to BPZ, the tissue cells may adapt to the toxicity and enhance the ability of antioxidant defense, and continue to produce a large amount of SOD. This may be due to the fact that the BPZ molecule has the largest number of carbon atoms, and there is a part of the carbon chain connected end to end, which leads to cell damage through the enzyme system, thus affecting the enzyme activity. In addition, in this research, the overall SOD activity of BPS did not change significantly, which can be seen as the SOD enzyme in zebrafish tissues was not seriously damaged after 13 days of BPs exposure, making ROS generation and elimination a state of dynamic balance. When Cheng et al. (2017) observed the sub-chronic toxicity of pyrrolidine to zebrafish, they also observed that the reduction of SOD activity might lead to the further accumulation of oxygen free radicals, making the body more sensitive to toxic substances [39].

**Figure 4:**
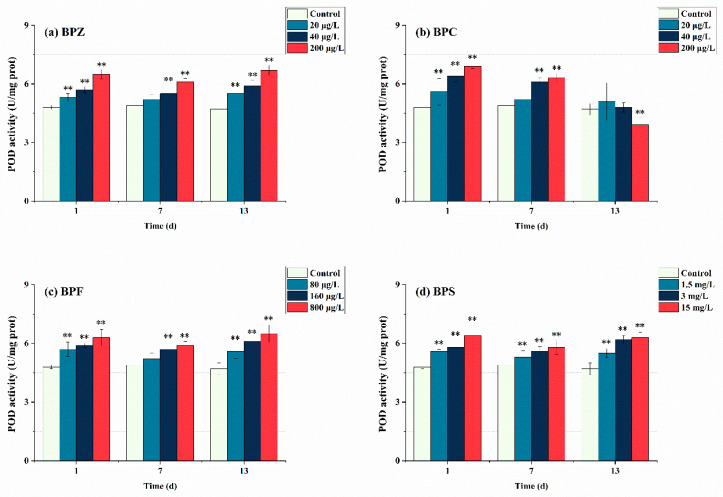
Effects of waterborne BPs exposure on the activity of POD in the tissues of zebrafish under different exposure conditions after 1, 7, and 13 days. The error bars represent the SD. The asterisks represent significant difference between treatment and control group at the same timepoint (*p* < 0.05, **).

As the second part of the antioxidant system, the detection of POD activity is equally important. As can be seen from Figure 4, after exposure to BPs for 1 day, POD in all treatments showed a trend of induction and had a significant effect (*p* < 0.05). This indicates that POD activity was induced at the initial stage of three BPs exposure, zebrafish produced a stress response after being poisoned, and POD activity increased to resist external adverse disturbance. This corresponds to SOD activity, and BPs induced many POD to remove H_2_O_2_ produced by the first line of defense. With the extension of exposure time, the BPZ, BPF, and BPS exposure treatments showed a trend of induction-inhibition-induction, and the overall POD activity was higher than the control group. This suggests that in the middle of exposure time, with the extension of BPs exposure time, BPs infestation seriously interferes with the antioxidant system of the fish body, and POD is not enough to eliminate the interference of pollutants, thus presenting a downward trend. In the later stage, zebrafish tissues adapted to BPs exposure, but there are still many ROS decomposed into H_2_O_2_ by SOD, so the enzymes in tissues continue to produce POD to cope with excessive H_2_O_2_. However, the situation in the BPC exposure treatment was completely different. After exposure to BPC for 1 day, POD activity in three treatments was inhibited. POD activity in the low-concentration treatment (20 μg/L) was close to that in the control group, while that in the medium and high-concentration treatments (40 μg/L and 200 μg/L) was still higher than that in the control group. When the time was extended to 13 days, the POD activity of medium and low concentration treatments (20 μg/L and 40 μg/L) was restored to the level of control group, while the POD activity of high concentration treatment (200 μg/L) was significantly lower than that of control group, and it had a significant effect (*p* < 0.05). These results indicate that the oxidative damage caused by BPC exposure at low and moderate concentrations to the tissue of zebrafish can achieve oxidation-antioxidant balance through self-regulation, and prolonged exposure to high concentrations of BPC may inactivate POD in zebrafish. From the perspective of chemical structure, the hydrogen atoms on the benzene ring of BPC may be replaced by methyl, leading to an increase in its irritation to the mucosa, resulting in an oxidative stress reaction. Mao et al. (2020) obtained a consistent result with this paper when studying the effect of strobilurin fungicides on POD activity in the liver of adult zebrafish [40].

**Figure 5:**
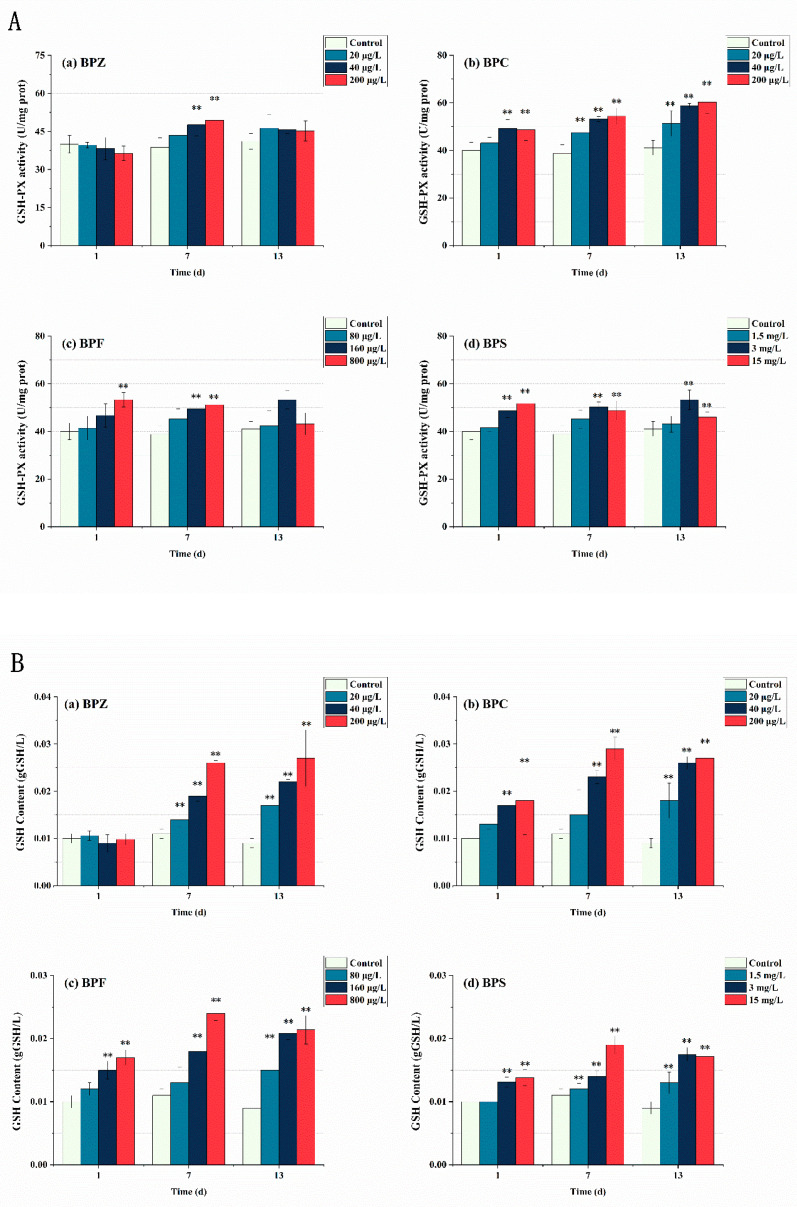
Effects of waterborne BPs exposure on the activity of GSH-PX (**A**) and the content of GSH (**B**) in the tissues of zebrafish under different exposure conditions after 1, 7, and 13 days. The error bars represent the SD. The asterisks represent significant difference between treatment and control group at the same timepoint (*p* < 0.05, **).

In this study, GSH-PX activity and GSH content in zebrafish tissues were also measured. As shown in Figure 5, GSH-PX activity increased significantly under BPs exposure. With the extent of exposure time, the number of BPs entering the fish body increases, inducing the synthesis of GSH to detoxify itself. Toxicity showed a time-effect relationship, and GSH content did not reach its maximum after 13 days of exposure. Interestingly, there was no significant change in GSH-PX activity and GSH content after exposure to BPZ for 1 day, indicating that zebrafish tissue had a slow stress response to BPZ exposure in terms of GSH. It can also be interpreted as oxygen free radicals induced by BPZ exposure being effectively cleared by other antioxidant enzymes. The results showed that the four BPs all induced GSH-PX activity and significantly increased the content of GSH. It is worth mentioning that GSH-PX activity and GSH content do not always correspond one to one, indicating that the generation of GSH is also related to other factors. Due to the limited exposure time selected in this experiment, the threshold at which GSH is induced is unknown.

### 3.5. mRNA Expression Levels of Genes Related to Oxidative Stress Response

**Figure 6:**
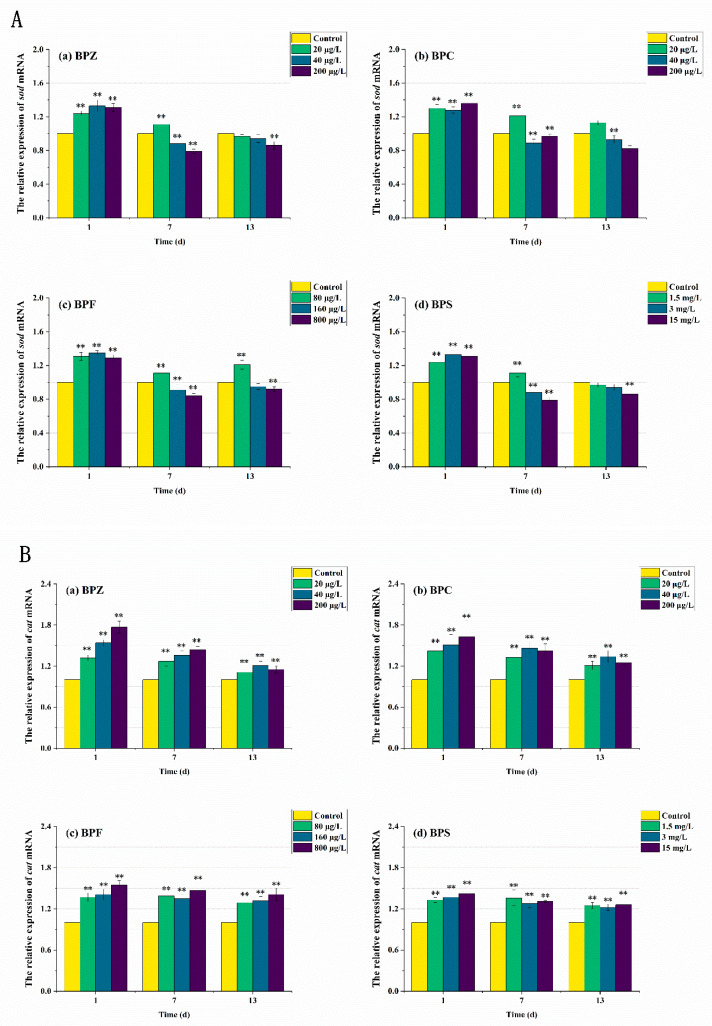

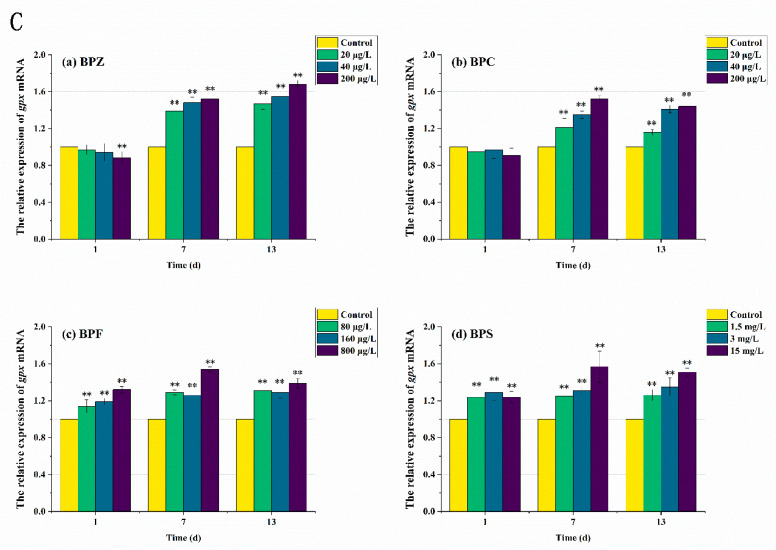
Effects of waterborne BPs exposure on mRNA levels of *sod* (**A**), *cat* (**B**), and *gpx* (**C**) in the tissues of zebrafish under different exposure conditions after 1, 7, 13 days. The error bars represent the SD. The asterisks represent significant difference between treatment and control group at the same timepoint (*p* < 0.05, **).

**Figure 7:**
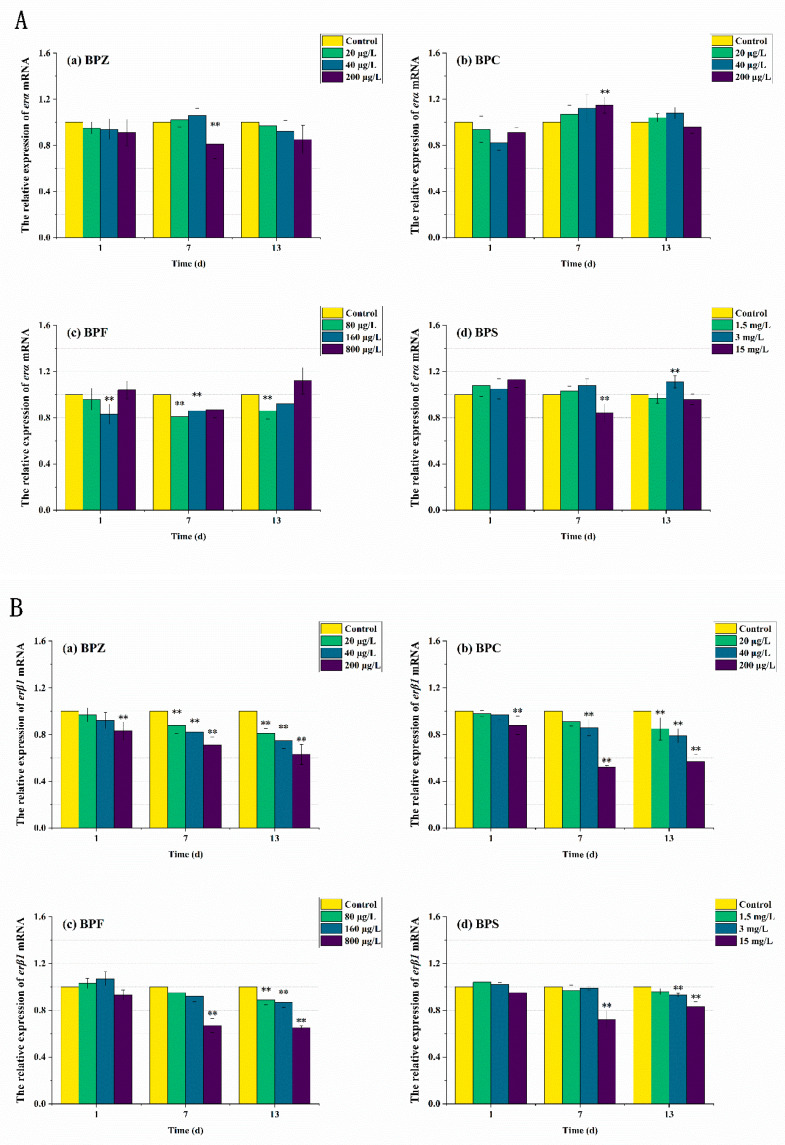
Effects of waterborne BPs exposure on mRNA levels of *erα* (**A**) and *erβ1* (**B**) in the tissues of zebrafish under different exposure conditions after 1, 7, 13 days. (*p* < 0.05, **).

In order to further elucidate the molecular mechanism of the antioxidant reaction, mRNA transcription levels after BPs exposure were also measured. As can be seen from Figure 6 and Figure 7, the expression levels of antioxidant-related genes and estrogen receptor (ER) genes in zebrafish were disturbed to varying degrees. The results showed that compared with the control group, the *sod* mRNA level was up-regulated to 1.3 times after the exposure to BPs for 1 day. When the exposure time was extended to the seventh day, the mRNA level of the medium-high concentration treatments fell below the normal level, while the mRNA level of the low-concentration treatment was still higher than the control group despite the down-regulation. After 13 days of exposure, *sod* mRNA levels in all exposed treatments except the BPC high concentration treatment (200 μg/L) were adjusted to about the normal value, which was basically consistent with the obtained SOD activity results. These results indicated that BPs altered the activity of these antioxidant enzymes by regulating gene transcription. However, we also observed a mismatch between the activity of antioxidant enzymes and the transcriptional levels of the genes encoding them. For example, after BPZ exposure (200 μg/L) for 13 days, *sod* mRNA in this treatment returned to the normal level, but SOD activity still increased, which could be explained by *cat* mRNA. As shown in Figure 6B, although the *cat* mRNA level of BPZ exposure treatment was inhibited at the later stage of exposure, it was still above the control level, indicating that a high concentration of BPZ exposure made zebrafish tissues produce more CAT to help SOD remove a large number of oxygen free radicals, so as to maintain the balance of the antioxidant system. The mRNA levels of *cat* and *gpx* in other groups were up-regulated on the first day, followed by slight fluctuations, but still up-regulated in general. This was also almost consistent with the enzyme activity, indicating that the *gpx* gene in zebrafish tissues was not sensitive enough to the exposure of BPZ and BPC or the reaction to stress was slow. However, with the extension of exposure time, the final mRNA level was increased to 1.4–1.6 times, much higher than that in the control group. Some recent studies have shown that changes in enzyme activity do not always correspond to changes in mRNA levels of related genes, possibly because an enzyme is composed of multiple gene clusters or because of time-lag effects between transcription and translation [41,42].

ER is a kind of ligand-activated transcription factor, which is a member of the nuclear receptor superfamily. The ER subtypes associated with humans are *erα* and *erβ*, which are encoded by different genes. ER binds to specific DNA sites directly or indirectly to exert transcriptional effects. *erα* and *erβ* have different biological effects. Ligand binding to *erα* promotes gene transcription, while binding to *erβ* inhibits gene transcription [43]. As can be seen from Figure 7, after 13 days of exposure to the four kinds of BPs, the relative expression level of *erα* in zebrafish tissues did not change significantly except for some high-concentration exposure groups. However, in BPZ, BPC, and BPF exposure groups, the relative expression level of *erβ_1_* in zebrafish tissues was significantly down-regulated in a concentration-effect relationship. These results suggest that the accumulation of BPs is related to the relative expression of ER in zebrafish. Interestingly, this was not significant in the BPS exposure group, which is almost consistent with the studies on anti-androgen and anti-estrogen effects in zebrafish [44].

## 4. Conclusions

The data measured in this study showed that after exposure to the four kinds of BPs, the tissues of zebrafish showed different levels of accumulation, forming a concentration–time effect, and did not reach the highest point of enrichment. Through the subacute toxicity test, the results showed that the exposure of BPZ, BPC, BPF, and BPS to water transport affected oxidative stress and the expression of oxidative-stress-related genes in zebrafish tissues to varying degrees. The four BPs all caused lipid peroxidation in the body. After 13 days of exposure, high concentrations of BPZ, BPC, BPF, and BPS led to a significant increase in MDA content in zebrafish tissues, which were 6.3, 5.9, 5.6, and 4.75 nmol/mg prot, respectively. Although the antioxidant defense system plays an important role in scavenging free radicals induced by BPs, it is not sufficient to balance the production and elimination of ROS. In addition, BPs exposure down-regulated the transcription levels of *sod* and up-regulated the transcription levels of *cat* and *gpx*, which were basically consistent with the results of oxidative stress. Furthermore, it was found that the relative expression level of *erα* in zebrafish tissues was not significantly changed by the four BPs, while the other three BPs significantly decreased the relative expression level of *erβ_1_* except BPS Complete results are hoped to contribute to the understanding of the mechanism of BPs induced subacute toxicity in zebrafish and provide a complement to the progress of toxicological research.

## Figures and Tables

**Table 1:** Design of concentrations of four BPs.

Name	1/100 LC_50_ (μg/L)	1/50 LC_50_ (μg/L)	1/10 LC_50_ (μg/L)
BPZ	20	40	200
BPC	20	40	200
BPF	80	160	800
BPS	1500	3000	15,000

**Table 2:** Primers used for qPCR analysis.

Gene	Sequence of the Primers (5′-3′)	Accession NO.	Size (bp)
*rp17*	F: CAGAGGTATCAATGGTGTCAGCCC R: TTCGGAGCATGTTGATGGAGGC	NM213213644.2	119
*β-actin*	F: CGAGCTGTCTTCCCATCCA R: TCACCAACGTAGCTGTCTTTCTG	AF025305.1	86
*erβ_1_*	F: GGG GAG AGT TCA ACC ACG GAG R: GCT TTC GGA CAC AGG AGG ACG	AJ414566	89
*erα*	F: CCC ACA GGA CAA GAG GAA GA R: CCT GGT CAT GCA GAG ACA GA	AF268283	250
*cat*	F: CTCCTGATGTGGCCCGATAC R: TCAGATGCCCGGCCATATTC	AF170069.1	126
*sod*	F: GTCCGCACTTCAACCCTCA R: TCCTCATTGCCACCCTTCC	BX055516	217
*gpx*	F: AGATGTCATTCCTGCACACG R: AAGGAGAAGCTTCCTCAGCC	AW232474	94

Note: F—Forward; R—Reverse.

**Table 3:** Linear ranges, linear equations, correlation coefficients (R^2^), LODs, LOQs, recovery rates and relative standard deviations (RSDs) of the four BPs.

Compound	Internal Standard Substance	Linear Range (μg/L)	Linear Equation	R^2^	LOD (μg/kg)	LOQ (μg/kg)	Recovery Rate (%)	RSD (%)
BPZ	BPA-^13^C_12_	1–100	y = 0.1044x + 0.0142	0.9998	0.60	1.89	87.92–96.13	2.84–16.36
BPC	BPA-^13^C_12_	1–100	y = 0.0478x – 0.1008	0.9989	0.12	0.38	85.98–100.10	4.63–11.83
BPF	BPA-^13^C_12_	1–100	y = 0.0568x – 0.4246	0.9994	0.16	0.54	83.30–98.23	1.47–8.63
BPS	BPS-^13^C_12_	0.5–100	y = 0.0616x + 0.3211	0.9990	0.019	0.06	92.79–102.91	4.27–14.74

## Data Availability

The authors declare that all data generated or analyzed during this study are included in the published article.
